# Supplementary material for: Reciprocal control of excitatory synapse numbers by Wnt and Wnt inhibitor PRR7 secreted on exosomes
Source: Nat Commun. 2018 Aug 24;9:3434. doi: 10.1038/s41467-018-05858-2 (PMC6109165; doi:10.1038/s41467-018-05858-2)
Supplement: Supplementary file 1 — Supplementary Information [file 41467_2018_5858_MOESM1_ESM.pdf]

Supplementary Information

Reciprocal Control of Excitatory Synapse Numbers by Wnt and Wnt Inhibitor PRR7 Secreted on Exosomes

Lee et al.

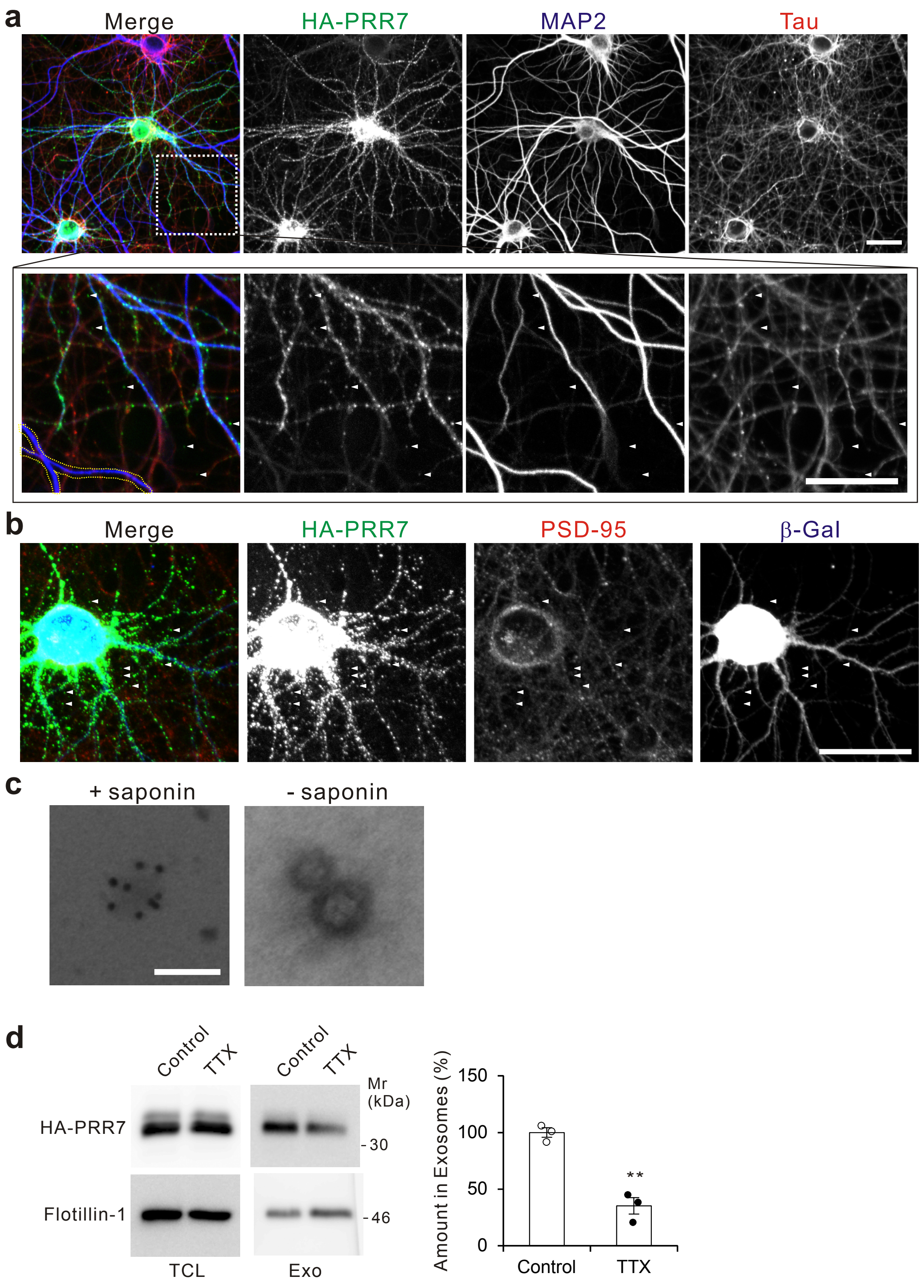

Supplementary Figure 1. Exosomal secretion of PRR7 by cultured rat hippocampal neurons.
(**a**) Top: immunofluorescent images of hippocampal neurons showing the extracellular presence of PRR7 (*green*)-positive speckles (marked by white arrowheads) outside the somatic dendritic and axonal areas of neurons which are stained by MAP2 (*blue*) and Tau1 (*red*) antibodies. Bottom: maginfied images of an area marked by dotted-line square box. Dendrites marked by yellow dotted line show diffuse dendritic staining of HA-PRR7 in Nxf neurons.(**b**) Immunofluorescent images of hippocampal neurons showing no colocalization of extracllular PRR7 speckles(marked by white arrowheads) with endogenous PSD-95. (**c**) ImmunoEM images of exosomes purified from the culture supernant of PRR7-transfected neurons, processed with or without membrane permeabilization (+ saponin or - saponin). (**d**) Effect of tetrodotoxin (TTX) on the exosomal secretion of PRR7. n = 3.Two tailed t-test: $^{**}p < 0.01$. Data are mean ± s.e.m. Scale bars, 20 μm (**a**,**b**) or 100 nm (**c**).

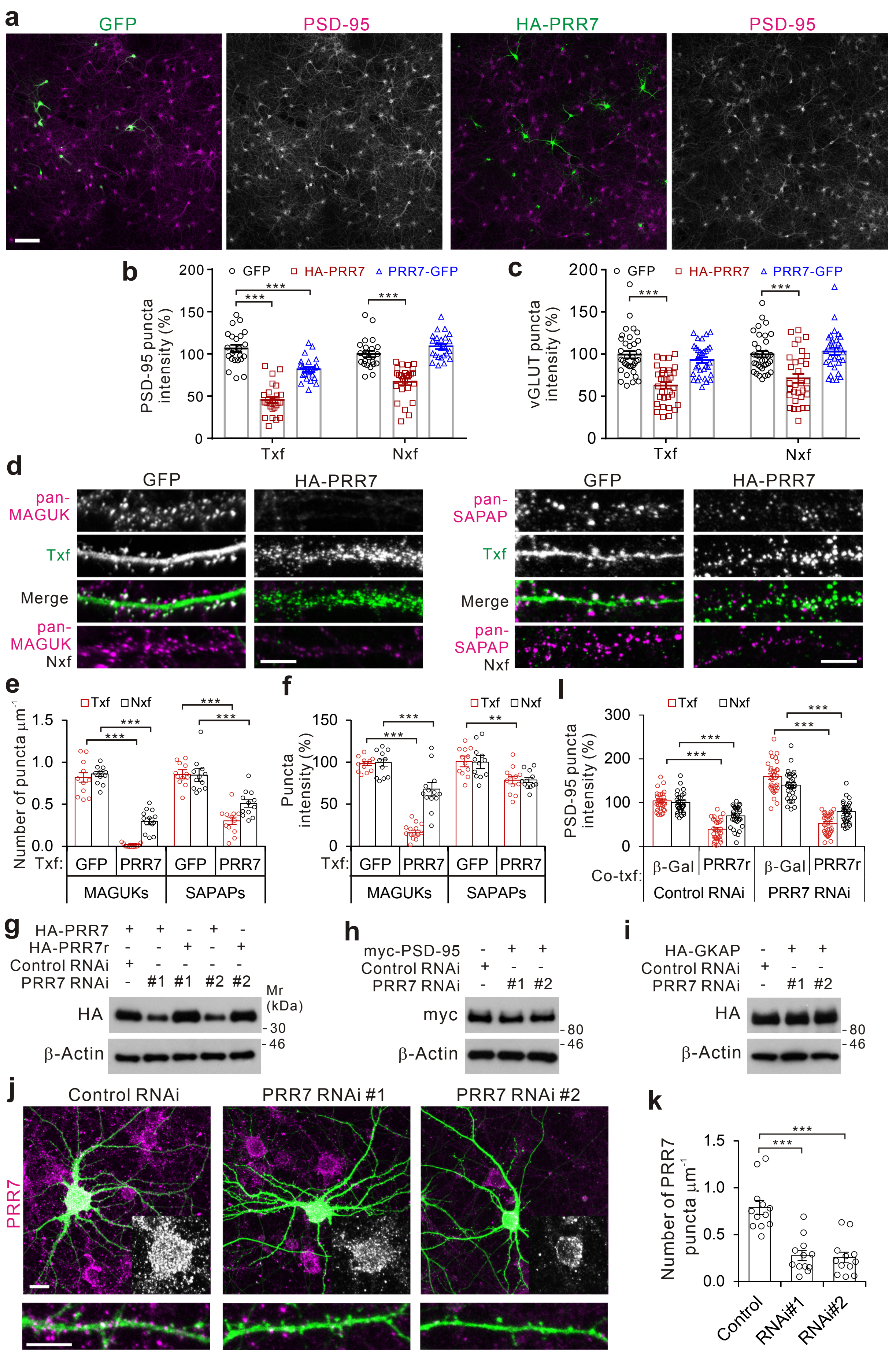

a
GFP
PSD-95
HA-PRR7
PSD-95
b
PSD-95 puncta intensity (%)
GFP
HA-PRR7
PRR7-GFP
Txf
Nxf
c
vGLUT puncta intensity (%)
GFP
HA-PRR7
PRR7-GFP
Txf
Nxf
d
GFP
HA-PRR7
pan-MAGUK
Txf
Merge
pan-MAGUK Nxf
pan-SAPAP
Txf
Merge
pan-SAPAP Nxf
e
Number of puncta µm-1
Txf
Nxf
Txf:
GFP
PRR7
GFP
PRR7
MAGUKs
SAPAPs
f
Puncta intensity (%)
Txf
Nxf
Txf:
GFP
PRR7
GFP
PRR7
MAGUKs
SAPAPs
l
PSD-95 puncta intensity (%)
Txf
Nxf
Co-txf:
β-Gal
PRR7r
β-Gal
PRR7r
Control RNAi
PRR7 RNAi
g
HA-PRR7
HA-PRR7r
Control RNAi
PRR7 RNAi
#1
#1
#2
#2
Mr (kDa)
HA
β-Actin
30
46
h
myc-PSD-95
Control RNAi
PRR7 RNAi
#1
#2
myc
β-Actin
80
46
i
HA-GKAP
Control RNAi
PRR7 RNAi
#1
#2
HA
β-Actin
80
46
j
Control RNAi
PRR7 RNAi #1
PRR7 RNAi #2
PRR7
k
Number of PRR7 puncta µm-1
Control
RNAi#1
RNAi#2

Supplementary Figure 2. Effect of PRR7 overexpression on excitatory synaptic markers and the specificity and efficacy of PRR7 RNAi and RNAi-resistant constructs.

(**a**) Immunofluorescent images of PSD-95 in hippocampal neurons transfected with either GFP (left) or HA-PRR7(right), acquired using a 10X objective lens. (**b**) Quantitation of PSD-95 puncta intensities in hippocampal neurons transfected with GFP, HA-PRR7, or PRR7-GFP. n = 23–25. Two-way ANOVA with Post-hoc Tukey's test: $F_{2,138}$ = 100.6, ***P < 0.0001. (**c**) Quantitation of vGLUT puncta intensities in hippocampal neurons transfected with GFP, HA-PRR7, or PRR7-GFP. n = 31–35, Two-way ANOVA, Tukey's test:$F_{2,190}$ = 37.98, ***P < 0.0001. (**d**) Representative immunofluorescent images of MAGUKs and SAPAPs in hippocampal neurons transfected with GFP or HA-PRR7. (**e**, **f**) Quantitation of MAGUK and SAPAP puncta densities (**d**) and intensities (**e**) in hippocampal neurons transfected with GFP or HA-PRR7. n = 11-13. Two-way ANOVA, Tukey's test: $F_{1,42}$ = 279 (MAGUKs density), $F_{1,44}$ = 90.59 (SAPAPs density),$F_{1,44}$ = 148.3 (MAGUKs intensity), $F_{1,44}$ = 23.27 (SAPAPs intensity),***P < 0.0001, **P = 0.0063. (**g**–**k**) Specificity and Efficacy of PRR7 knockdown constructs. Effect of PRR7 RNAi on the protein levels of HA-PRR7 (**g**), Myc-PSD-95, and HA-GKAP (**i**) expressed in COS7 cells. Representative images of hippocampal neurons showing the effect of PRR7 RNAi on endogenous PRR7 protein levels (**j**) and quantification of PRR7 puncta numbers in the dendrites (**k**). n = 12. One-way ANOVA, Tukey's test:$F_{2,33}$ = 24.08, ***P < 0.0001. (**l**) Quantitation of PSD-95 puncuta intensities in the PRR7 RNAi rescue experiments with HA-PRR7r. n = 31 each. Two-way ANOVA, Tukey's test:$F_{3,240}$ = 164, ***P < 0.0001. Scale bars, 5 μm for all images except 100 μm for (**a**). Data are mean ± s.e.m.

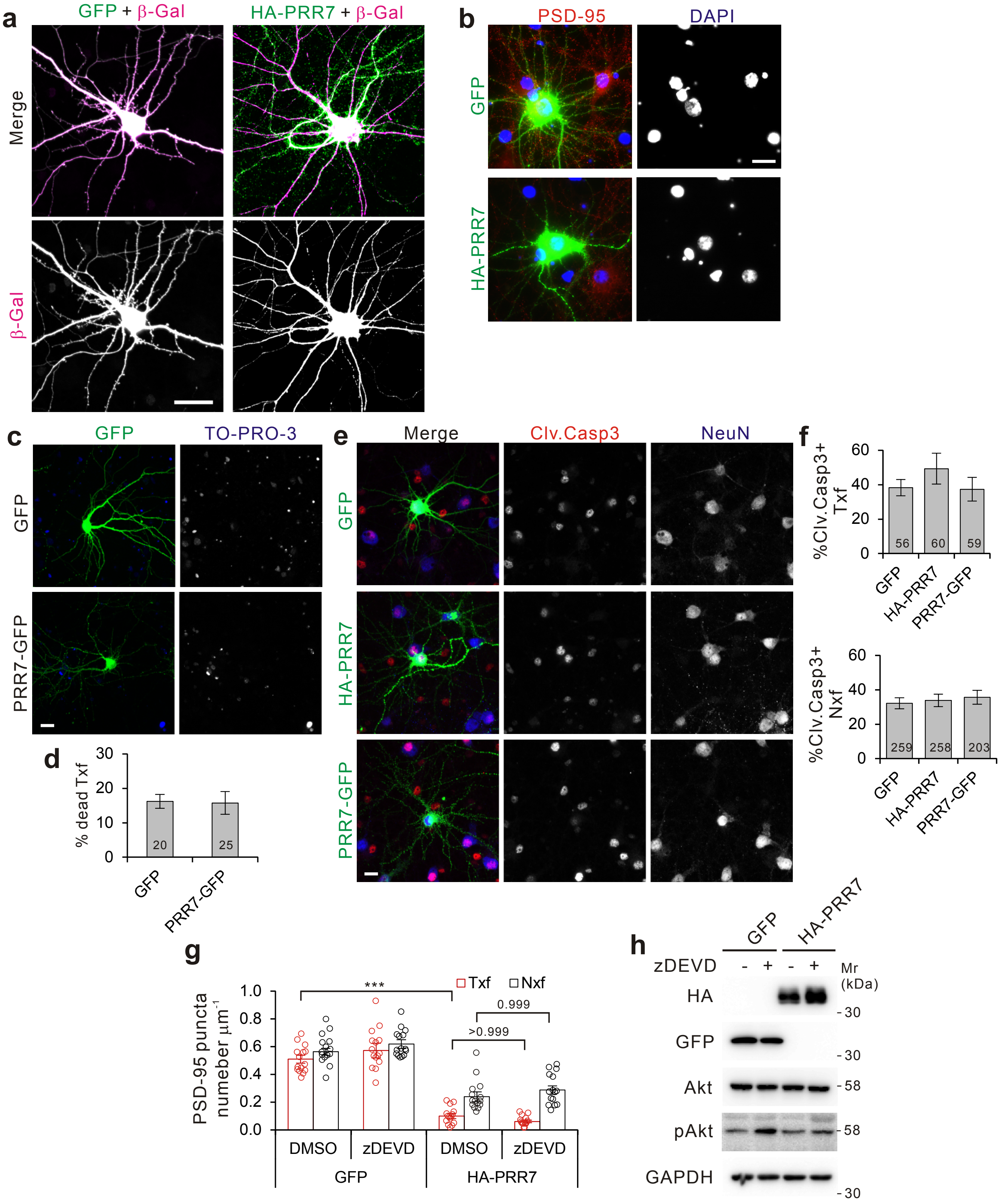

Supplementary Figure 3. PRR7 overexpression for 24 h does not induce significant neuronal death. (**a**) Normal denderitic tapering and arborization of PRR7-transfected neuron in comparison to GFP-transfected neuron. (**b**)Representative images of PSD95 and DAPI staining of hippocampal neurons transfected with GFP or HA-PRR7. (**c**,**d**) TO-PRO3 dye exclusion tests. Representative fluorescent images of GFP- or PRR7-GFP-transfected hippocampal neurons stained with the dye (**c**) and quantitation of the data (**d**). (**e**,**f**) Cleaved caspase 3 staining of hippocampal neurons transfected with GFP, HA-PRR7, or PRR7-GFP. Representative immunofluorescent images (**e**) and quantified data (**f**). Numbers in the bar graphs represent the numbers of neurons analyzed. (**g**) Quantitation of z-DEVD-FMK (zDEVD) effect on PSD-95 puncta density in hippocampal neurons transfected with GFP or HA-PRR7. n = 15 per condition.Two-way ANOVA with post-hoc Sidak's test: $F_{3,112}$ = 158.7, ***P < 0.0001. (**h**) Western blotting analyses of zDEVD effect on Akt and pAkt levels in hippocampal neurons transfected with GFP- or HA-PRR7. Scale bars, 20 μm. Data are mean ± s.e.m.

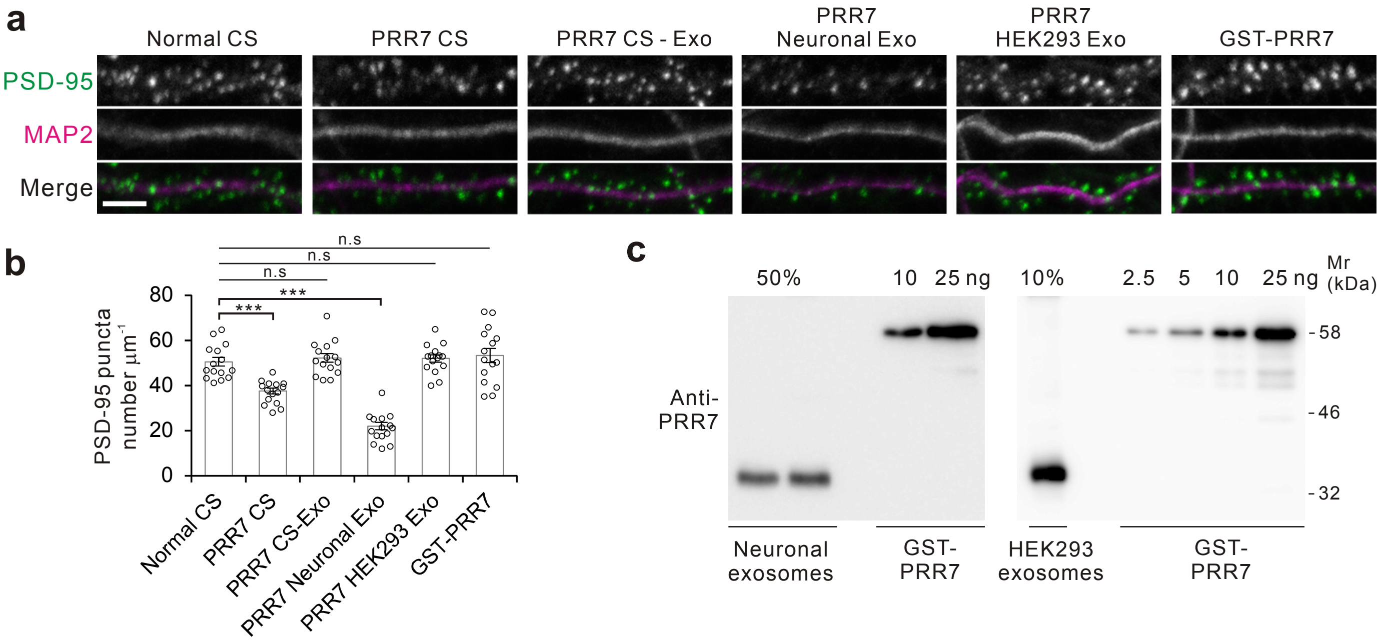

Supplementary Figure 4. Effect of PRR7 CS and exosomes on PSD-95.
(**a**) Representative images of dendrites showing the effect of various CS, exosomes, and purified GST-PRR7 protein (250 ng) on PSD-95 clusters in hippocampal neurons. Scale bar, 5 μm. (**b**) Quantification of the effect on the PSD-95 puncta density. n = 15 from 2 replicates. One-way ANOVA with post-hoc Tukey's test: $F_{5,84}$ = 39.04 , ***p < 0.001. Data are mean ± s.e.m. (**c**) Semi-quantitaive wersten blot analyses of PRR7 amounts in exosomes purified from HA-PRR7 transfected neurons or HEK293 cells. Purified GST-PRR7 proteins were used as calibration standards.% indicates gel-loading amounts of exosomes normalized to the amount used for treating neurons in **a**.

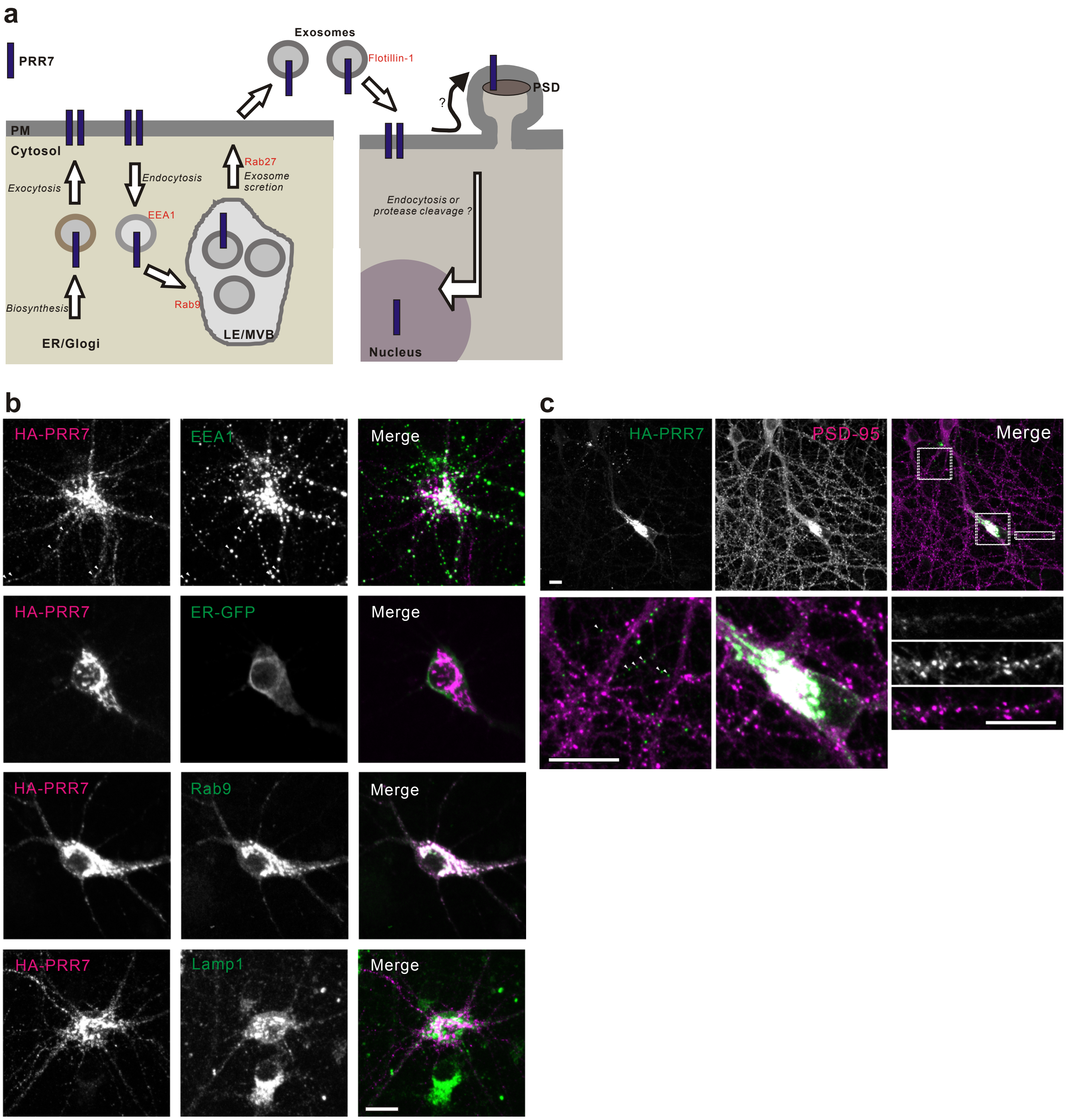

Supplementary Figure 5. Endosomal trafficking model of PRR7.
(**a**) Schematic model depicting the endosomal trafficking of PRR7.(**b**) Representative immunofluorescent images of HA-PRR7 co-stained with various markers of endosomal membranes in hippocampal neurons. White arrowheads in top panels indicate co-localized HA-PRR7 with EEA1. (**c**) Immunofluorescent staining images of HA-PRR7 and PSD-95 in hippocampal neurons 3 h post-transfection. White arrowheads indicate exosome-localized HA-PRR7. Boxed areas (with white dotted line) indicate blow-up images shown in the bottom low. Scale bars, 10 μm.

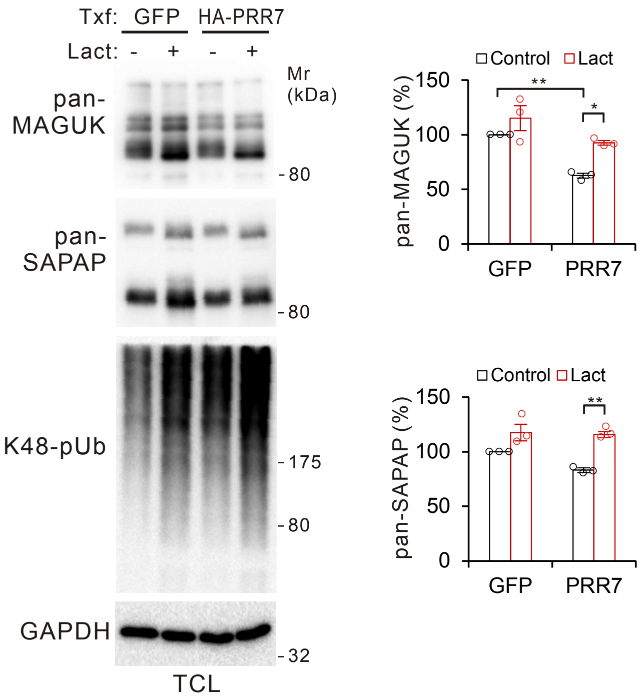

Supplementary Figure 6. HA-PRR7 induces the proteasomal degradation of MAGUKs and SAPAPs. Representative immunoblots showing the effect of Lact on the total protein levels of MAGUKs and SAPAPs in hippocampal neurons transfected with GFP- or HA-PRR7. n = 3. Two-way ANOVA with post-hoc Tukey's test: $F_{1,8}$ = 14.61 (MAGUKs), $F_{1,8}$ = 36.48 (SAPAPs). **P =0.0091, *P = 0.0291, and **P = 0.0024 (SAPAPs). Data are mean ± s.e.m.

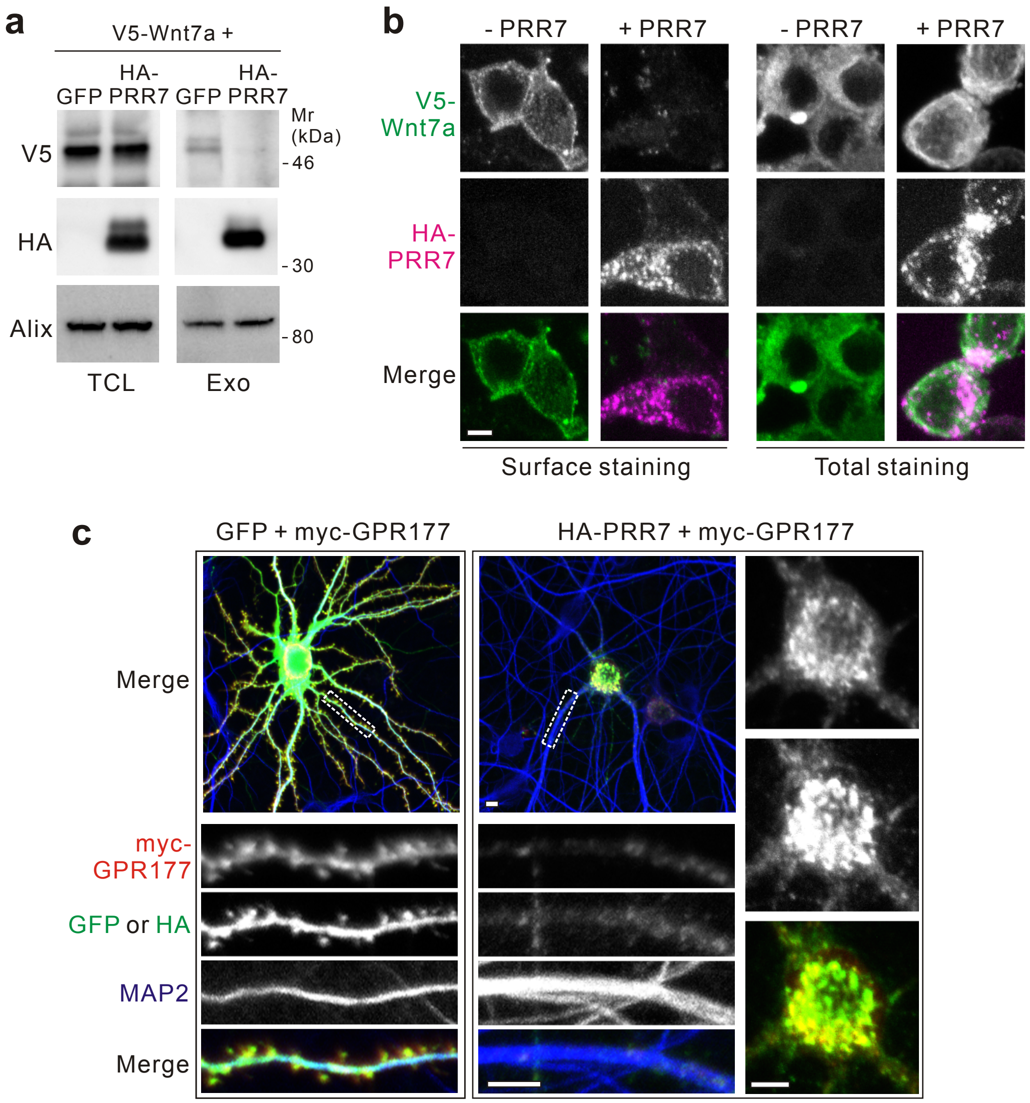

Supplementary Figure 7. PRR7 affects the exosomal secretion and surface expression of Wnts and alters the subcellular localization of GPR177.

(**a**) PRR7 overexpression inhibits the exosomal secretion of Wnt7a in cultured hippocampal neurons. (**b**) Representative immunofluorescent images of HEK293 cells showing the effect of PRR7 overexpression on the surface expression of Wnt7a. Total staining shows comparable expression of V5-Wnt7a in cells regardless PRR7 co-transfection. (**c**) Representative immunofluorescent images of cultured hippocampal neurons showing the effect of PRR7 overexpression on the subcellular localization of GPR177. Scale bars, 5 μm.

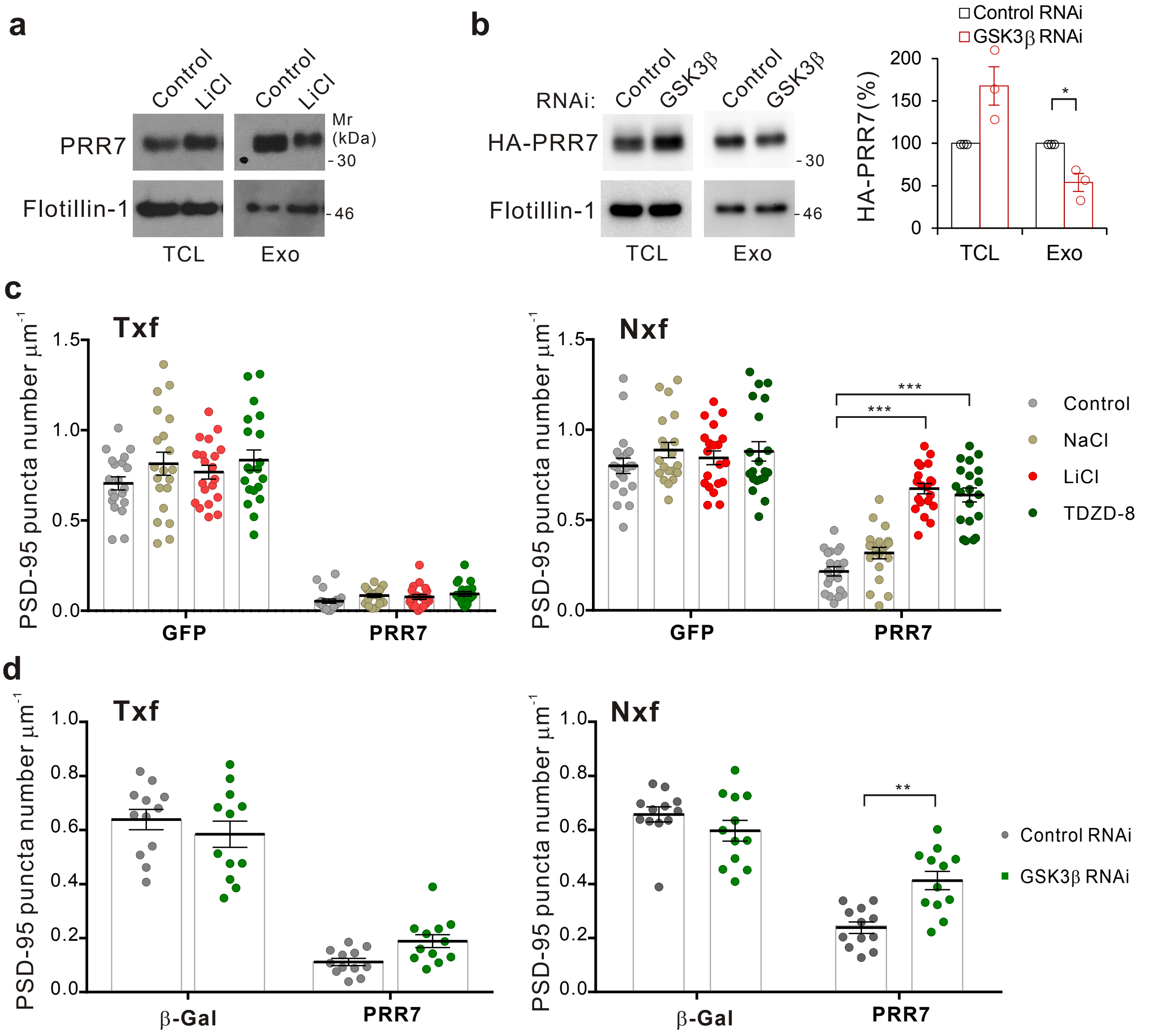

Supplementary Figure 8. GSK3β activity is required for the exosomal secretion of PRR7.
(**a**) Representative immunoblots showing the effect of LiCl on the exosomal secretion of HA-PRR7. n = 3. (**b**) Effect of GSK3β shRNAs on the exosomal secretion of HA-PRR7. Representative immunoblots and quantification of the data, normalized to flotillin-1 levels. n = 3. Two-tailed unpaired t-test with Welch's correction: $t_2 = 4.378$, *P = 0.0484. (**c**) Quantification of the effect of GSK3 inhibitors on the PRR7-induced loss of PSD-95 in hippocampal neurons. Two-way ANOVA, Post-hoc Tukey's test: $F_{3,152} = 20.5$, ***$P < 0.0001$. (**d**) Quantification of the effect of GSK3 shRNAs on the PRR7-induced loss of PSD-95 in hippocampal neurons. Two-way ANOVA with post-hoc Tukey's test: $F_{1,44} = 3.393$,**$P = 0.0014$. Data are mean ± s.e.m.

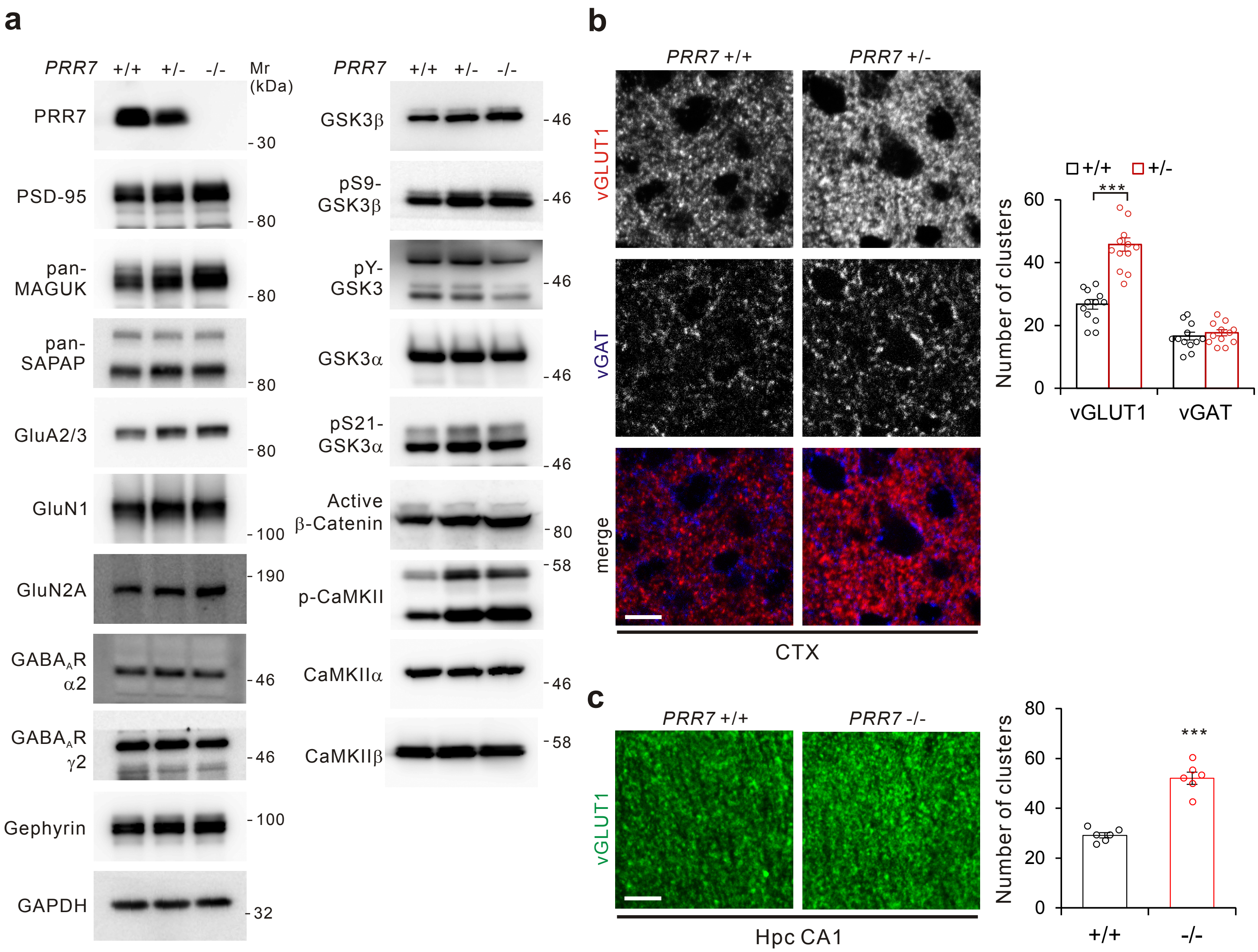

Supplementary Figure 9. *PRR7* knockout mice show increased number of excitatory synapses.
(**a**) Representative western blots showing the relative protein levels of indicated proteins in the hippocampi of 2-month old *PRR7* WT (+/+), heterozygote (+/-) and homozygote knockout (-/-) mice. n =2 mice per genotype. (**b**) Representative images of vGLUT and vGAT immunohistochemistry in the layer 2/3 cortices (CTX) of *PRR7* WT and heterozygote knockout mice (top panels) and quantification of the puncta densities in 25 x 25 μm area. n = 12 from 3 animals per condition. Two-way ANOVA with post-hoc Tukey's test: $F_{1,44} = 42.05$, ***p < 0.0001. (**c**) Representative images of vGLUT immunohistochemistry and quantification of puncta densities in the stratum radiatum of hippocampal CA1 (Hpc CA1). n = 6 from 2 animals. t-test, ***p < 0.001. Data are mean ± s.e.m. Scale bar, 10 μm.

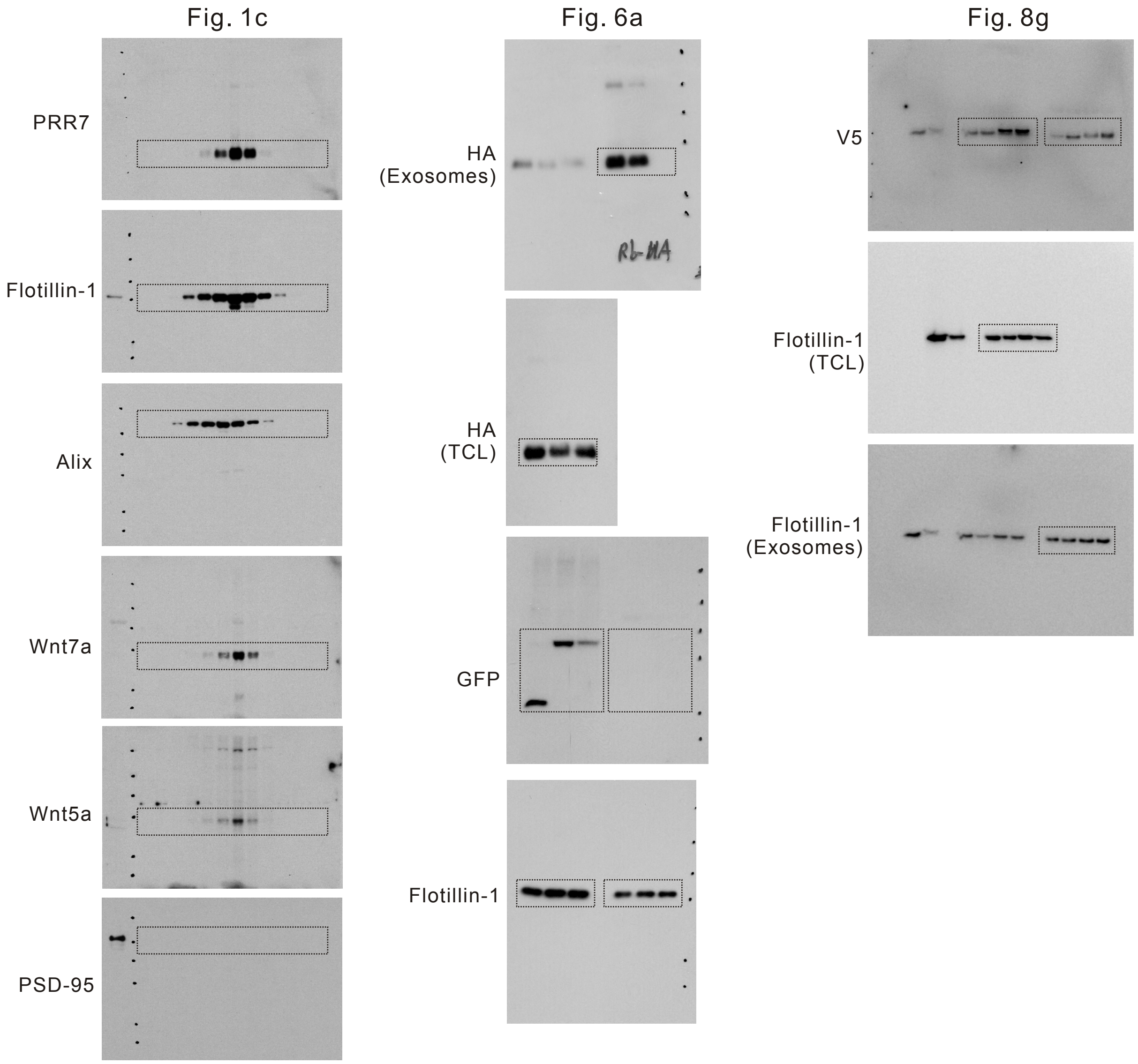

Supplementary Figure 10. Uncropped scans of the most important blots.
